# Mid-term clinical and sonographic outcomes of minimally invasive acromioclavicular joint reconstruction: mini-open versus arthroscopically assisted

**DOI:** 10.1007/s00402-023-05110-7

**Published:** 2023-11-08

**Authors:** A. Behrens, P. Behrendt, M. J. Heintzen, J. Finn, A. Seekamp, K. Mader, S. Lippross, T. O. Klatte

**Affiliations:** 1 Department of Trauma and Orthopaedic Surgery, University Medical Center Hamburg-Eppendorf, Martinistrasse 52, 20251 Hamburg, Germany; 2 Asklepios Hospital St. Georg, Hamburg, Germany; 3 Department of Anatomy, Kiel University, Kiel, Germany; 4 Orthopraxis Kiel, Kiel, Germany; 5 Department of Trauma and Orthopedic Surgery, University Medical Center Schleswig-Holstein (UKSH), Kiel, Germany

**Keywords:** AC joint dislocation, AC joint stabilization, Acute

## Abstract

**Introduction:**

The current literature describes various operative stabilization strategies which achieve good clinical outcomes after acute acromioclavicular joint (ACJ) dislocation. The aim of this study was to compare the mid-term clinical and sonographic treatment outcomes after minimally invasive mini-open and arthroscopic reconstruction.

**Materials and methods:**

We conducted a retrospective two-center study of patients with acute ACJ dislocation. Surgical treatment was performed using either a mini-open approach (MIOP) or an arthroscopic technique (AR). The primary outcome parameters of this study were the sonographically measured acromioclavicular (ACD) and coracoclavicular distances (CCD). Secondary outcome parameters included the Constant–Murley score (CS), range of motion (ROM), postoperative pain scale (VAS), return to daily routine, return to sports, complications, as well as operative revisions.

**Results:**

After a mean follow-up of 29 months, 30 patients were included in this study with an average age of 41.3 ± 14.8 years (MIOP) and 41.2 ± 15.4 years (AR). The sonographic ACD (MIOP 9.11 mm vs. AR 8.93 mm, *p* = 0.41) and CCD (MIOP 25.08 mm vs. AR 24.36 mm, *p* = 0.29) distances showed no statistically significant differences. Furthermore, there was no statistically significant difference when compared to the contralateral side (*p* = 0.42). With both techniques, patients achieved excellent clinical outcome parameters without statistically significant differences in CS (MIOP 95 vs. AR 97, *p* = 0.11) and VAS (MIOP 1.76 vs. AR 1.14, *p* = 0.18). The return to daily activity and return to sport rates did not differ. There were neither complications nor revisions in both groups.

**Conclusion:**

Both minimally invasive techniques for acute ACJ stabilization achieved excellent clinical and sonographic outcomes without one technique being statistically superior to the other.

## Introduction

Acute acromioclavicular joint (ACJ) dislocation is a common injury, making up approximately 17% of all shoulder injuries [1, 2]. In a recent prospective registry study, ACJ injuries accounted for 54% of sports-related acute shoulder injuries, with a peak in patients aged 15–30 years old [3]. ACJ dislocations are defined as “acute” when occurring within 3 weeks after trauma, which plays a very important role when deciding treatment strategy [4]. Delayed treatment has been identified as a negative prognostic factor and may necessitate biological augmentation [5]. The most widely used classification is according to Rockwood et al. with the ISAKOS statement modification [6, 7]: Rockwood type I and II injuries are low-grade dislocations with a conservative treatment recommendation, whereas Rockwood type IV to VI injuries are high-grade dislocations which require a surgical ACJ reconstruction (ACJR). The management of Rockwood type III dislocations remains under debate; however, the surgical treatment of a type IIIB injury in a patient with a high functional demand should be considered, since dynamic horizontal instability has been shown to be an independent risk factor for poor functional outcome [7–9].

Over the years, many different operative stabilization techniques have been described for acute ACJ dislocations; nevertheless, internal fixation with a hook plate as well as minimally invasive techniques using a pulley system are seen as the current standards of care [9, 10] Regarding the minimally invasive techniques, mainly two CC stabilization techniques have been investigated—a single or double-tunnel technique. Both techniques can be combined with additional horizontal stabilization via an AC cerclage in high-grade ACJ dislocations [11, 12]. The main difference between the minimally invasive techniques is the approach, namely, an arthroscopic versus mini-open approach. Both techniques have been reported to achieve good to excellent outcomes, but comparative analyses are sparse. A potential advantage of the arthroscopic technique is the detection of intra-articular pathologies. On the other hand, the mini-open technique is easily performed by a trauma surgeon without arthroscopic surgery expertise and without the need for arthroscopic surgery equipment.

The main scope of this study was to compare the clinical and sonographic outcomes of ACJ dislocation treatment with the minimally invasive mini-open and arthroscopic reconstruction techniques. We hypothesized both techniques to result in equal clinical and sonographic results at mid-term follow-up.

## Materials and methods

### Study design

From 2018 until 2020, 30 patients with acute ACJ dislocations were included in a retrospective two-center two-surgeon study (University Medical Center Hamburg Eppendorf, University Medical Center Schleswig-Holstein). The study design was approved by the local ethics committee and informed consent was obtained from each patient (D 418/16).

The main inclusion criteria included a Rockwood type ≥ IIIb acute ACJ dislocation which was treated with a mini-open technique (MIOP) at one study center and an arthroscopic technique (AR) at the other center. Vertical instability was measured in an AP view of both AC joints with a 5 kg load. Dynamic horizontal instability (type IIIb) was defined by bilateral Alexander view radiographs according to the ISAKOS consensus statement [7]. Minimum follow-up time was defined as 24 months and patient age between18 and 60 years. Exclusion criteria included all chronic ACJ dislocations, polytrauma patients, or any major upper limp injury as well as missing informed consent. A preoperative MRI was mandatory in the MIOP group and optional in the AR group.

Applying the inclusion und exclusion criteria, 45 out 100 patients operated during the specified period were excluded. Among the 55 patients, 25 could not be included in this study resulting in a loss of follow-up of 45%.

### Operative technique and postoperative rehabilitation protocol

**Fig. 1:**
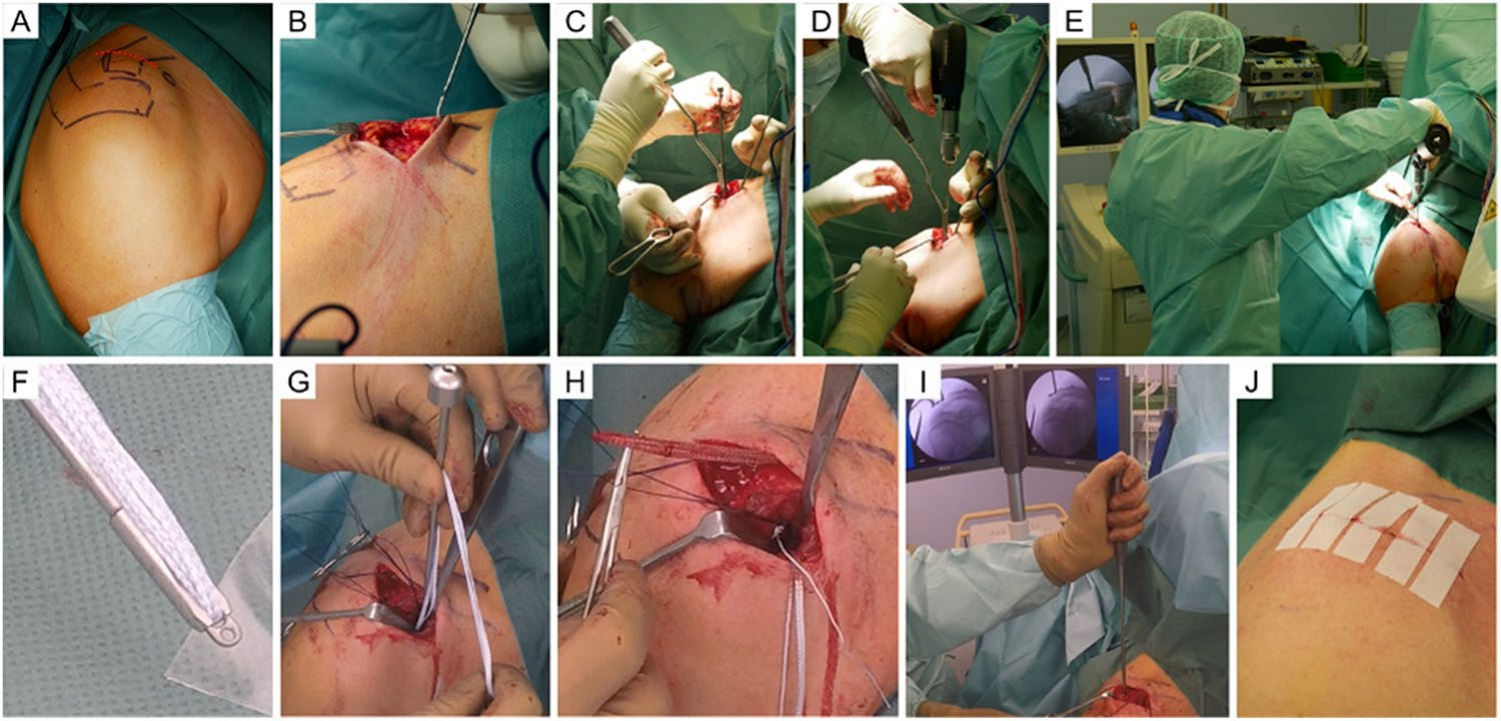
Mini-open technique for acute ACJR. **A** A 5 cm parasagittal skin incision is made to expose the trapezius-deltoid fascia and lateral aspect of the clavicle (**B**). The coracoid process is exposed by a delta split anterior to the clavicle and a drill guide is placed from laterally underneath the coracoid base (**C**). Positioning of a drill pin in the center of the coracoid base (**D**). Under fluoroscopic control, the drill pin is over-reamed with a 4.5 mm drill (**E**). Using an insertion guide, an Endobutton (Storz) is loaded with two Fibertapes (Arthrex Inc.) and is then flipped at the inferior margin of the coracoid base (**F**, **G**). Both sutures are shuttled through the medial and lateral drill holes in the clavicle to create a Y-shape construct (**H**). The lateral clavicle is reduced using a reduction guide and both fiberwire sutures are knotted on to an Endobutton (Storz) at least seven times (**I**). The ACJ joint capsule is reconstructed using vicryl sutures and the skin is closed with absorbable suture material (**J**)

The modified MIOP technique (MINAR^®^, Storz, Fig. 1) is based on the technique described by Petersen et al. [13].

The patient was placed in a beach chair position. A 5 cm parasagittal skin incision was made. After identification of the ACJ using a needle, the clavicular part of the deltoid muscle was split and the coracoid process was exposed. A drill guide was placed underneath the coracoid at its posterior “knee” to place a central k-wire. Afterward, using the k-wire as a guide, a hole was reamed with a 4.5 mm cannulated drill. Another guide was used to place an Endobutton (Storz) loaded with two Fibertape sutures (Arthrex Inc.) underneath the coracoid. Correct subcoracoid flipping of the button was controlled fluoroscopically. Afterward, two 2.5 mm central tunnels were drilled close to the tuberosity in the clavicle, with one more lateral and the other more medial. Both Fibertape sutures were then shuttled through the clavicle. After manual reduction using a specific reduction aid, the sutures were fixed onto an Endobutton (Storz) and secured with 7 knots. Minimal vertical overreduction was aimed for due to known postoperative loss of reduction. The acromioclavicular joint capsule and the trapezius-deltoid fascia were closed with vicryl sutures. An arthroscopy was only performed in patients with concomitant intra-articular injuries. SLAP lesions were treated with debridement and labral tears were fixated using two suture anchors.

**Fig. 2:**
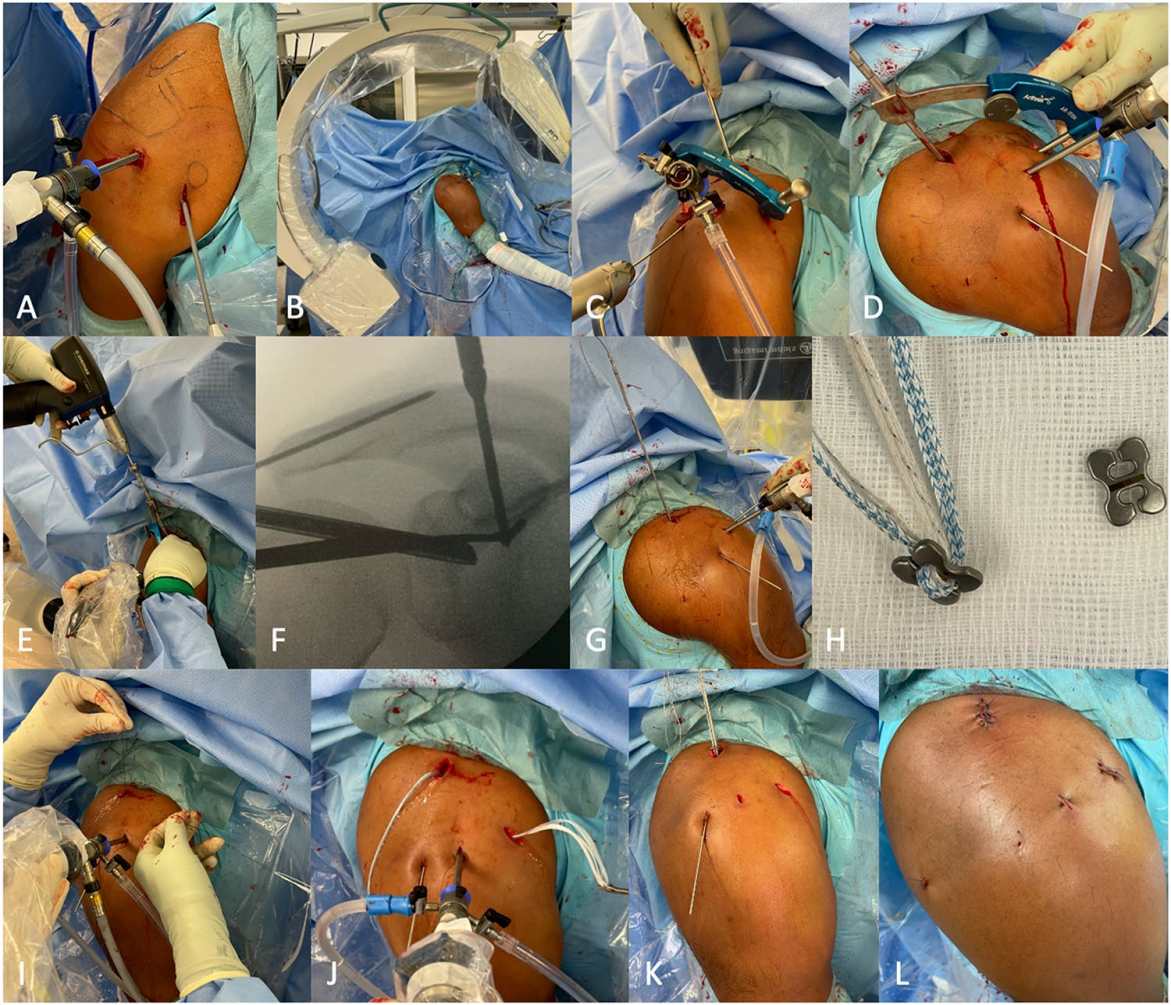
Arthroscopic-assisted technique. **A** After a standard diagnostic arthroscopy, the base of the coracoid is prepared via the anterior portal. **B** Under radiographic control, the ACJ is reduced and temporarily fixed with a K-wire (**C**). **D** Via the anterior porta, a guide instrument is placed underneath the coracoid base and via radiographic control on top of the clavicle. Using the guide instrument, transclavicular and transcoracoidal holes are drilled under radiographic and arthroscopic controls (**F**). **G** A guide wire is shuttled through the drill guide, the drill guide is then removed. **H** The pulley system (DogBone® attached to a TightRope®) is shuttled with the guide wire and placed at the coracoid base under arthroscopic control (**I**, **J**). **K** With another DogBone® Button, the vertical stabilization is secured. **L** Finally, an additional AC cerclage can be placed with the remaining K-wire. Afterward, the skin is closed with a monofilament suture

The arthroscopic-assisted technique (DogBone^®^, Arthrex, Fig. 2) was also performed in beach chair position.

After a diagnostic arthroscopy of the affected shoulder, the coracoid base was visualized and prepared via the anterior and anterolateral portals. Afterward, a 1 cm incision was made superior to the clavicle at the height of the linea trapezoidea. Under radiographic control, the ACJ was reduced and temporarily fixed with K-wires. Afterward, single-tunnel transclavicular and transcoracoidal holes were drilled using an aiming guide. After radiographic control, a pulley system was inserted (DogBone^®^ attached to a TightRope^®^, Arthrex Inc.) and fixed with another DogBone^®^ button. Using TightRope^®^ sutures, additional horizontal AC cerclage was performed in every case as described in the previous paragraph [14].

Postoperative treatment was administered according to a standard protocol after ACJ stabilization. All patients were treated with a brace immediately after surgery for 6 weeks. Active-assisted range of motion up to 60° of abduction/flexion was allowed immediately after surgery for 3 weeks and up to 90° in the following 3 weeks. Afterward, range of motion was unlimited, but muscle strengthening exercises were paused up to 10–12 weeks. Competitive athletes and patients with a high functional demand were first allowed to return to sports 3 months after surgery.

### Outcome assessment

**Fig. 3:**
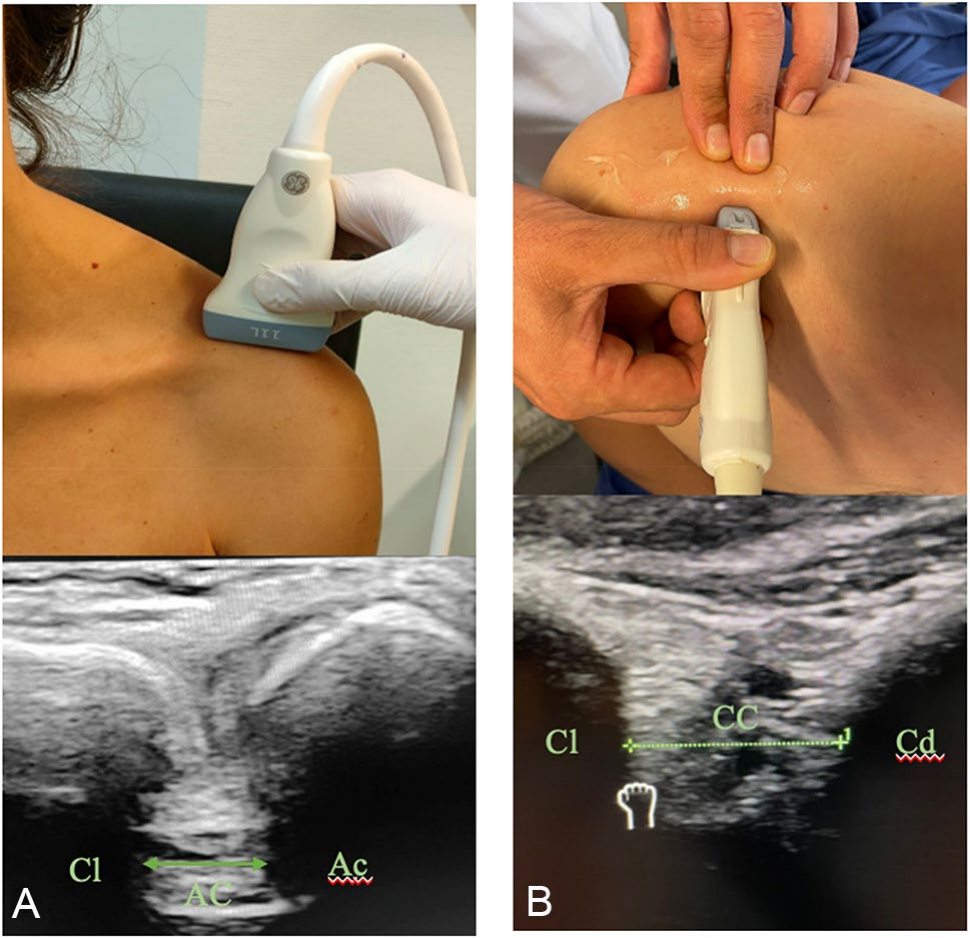
Sonographic assessment of AC (**A**) and CC (**B**) distance following ACJ reconstruction. *Cl* clavicle, *AC* acromioclavicular distance, *Ac* acromion, *CC* coracoclavicular distance, *Cd* coracoid

Follow-up examinations were conducted at least 24 months following initial surgery and incorporated functional outcome scoring systems including the Constant–Murley score (CS) and subjective pain by visual analog scale (VAS). Range of motion (ROM) and return to sports was recorded (0: no sports possible–4: return to pre-injury sport level). In addition, sonographic acromioclavicular (ACD) and coracoclavicular distances (CCD) were measured on the injured and contralateral sides (illustrated in Fig. 3). The relation to the contralateral side was recorded (side-to-side difference, SSD). Furthermore, possible complications and/or operative revisions were noted.

### Statistical analysis

The data are presented as means and standard deviations (SD). The primary outcomes were defined by the sonographic measurements. Secondary outcomes were CS, VAS, ROM, and return to sport/activity. Differences between the groups were calculated with the student’s *t*-test and the Kruskal–Wallis test for non-parametric parameters. Categorical parameters were compared using the chi-squared test, with the Fisher’s exact text used for categorical parameters in the case of small subgroups (*n* < 5). Statistical analysis was performed using GraphPad Prism 8 (San Diego, CA, US). A *p* value < 0.05 was considered significant. A sample size calculation revealed *n* = 30 patients using G-Power (version 3.1, Institut für Experimentelle Psychologie, Heinrich Heine Universität, Düsseldorf, FRG) with an α-error of 5% and test power of 0.80, with 10 points in the CS considered a meaningful difference [5, 15].

## Results

### Patient demographics

30 patients with acute ACJR were included in this study with a mean follow-up of 29 ± 11.16 months (Table 1). There were no demographic differences between the MIOP and AR groups (*p* > 0.05).

**Table 1:** Demographic data (MIOP mini-open ACJR; AR arthroscopic ACJR; SLAP superior labral tear from anterior to posterior)

	MIOP (*n* = 16)	AR (*n* = 14)
Sex (male/female)	15/1	13/1
Age (years)	41.3 ± 14.8	41.2 ± 15,4
Follow-up (months)	30.2 ± 9.96	27 ± 8.43
Dominant side	9 (62.5%)	7 (50.0%)
Rockwood classification		
▪ Rockwood type IIIb	6 (37.5%)	4 (28.6%)
▪ Rockwood type IV	3 (18.7%)	3 (21.4%)
▪ Rockwood type V	7 (43.7%)	7 (50.0%)
Time until surgery (days)	7.7 ± 5.0	6.6 ± 3.6
Preoperative MRI	16	0
Concomitant injury		
▪ Bankart lesion	1	0
▪ Rotator cuff tear	0	0
▪ SLAP lesion	1	2
▪ Fracture	0	0
Surgical time (min)	74.3 ± 23.2	86.3 ± 28.4

### Radiographic outcomes

The sonographic measurements revealed no significant side-to-side CCD (MIOP 1.1 ± 0.1 mm vs. AR 1.0 ± 0.8 mm, *p* = 0.42) and ACD (MIOP 1.3 ± 0.6 mm vs. AR 1.1 ± 0.8 mm, *p* = 0.09) differences between the two study groups. In addition, tunnel positioning was evaluated in the immediate postoperative radiographs. Using the MIOP technique, the distance of the lateral and medial tunnels from the medial ACJ were 26.8 ± 4.1 and 47.3 ± 6.4, respectively. In addition, radiographs were available in the MIOP group at follow-up. There was a significant loss of reduction compared to the immediate postoperative radiographs (MIOP day 1 postoperative 3.43 ± 1.31 mm vs. follow-up 5.4 ± 1.92 mm, *p* < 0.0001), but there was no significant difference when compared to the contralateral side (4.88 ± 1.54 mm, *p* = 0.99). The CCD showed a non-significant increase by follow-up (MIOP day 1 postoperative 6.33 ± 3.33 mm vs. follow-up 8.26 ± 4.37 mm, *p* = 0.1488) without significant difference to the contralateral side (10.40 ± 3.05 mm, *p* = 0.1442). Only immediate postoperative radiographs were available in the AR group; the distance of the tunnel was 7.21 ± 3.92 mm medial to the ACJ.

### Clinical outcome scores

There were no significant differences between both techniques in terms of CS (MIOP 95.0 ± 14.9 vs. AR 97.0 ± 3.0, *p* = 0.11) and VAS (MIOP 1.76 ± 2.3 vs. AR 1.1 ± 1.86, *p* = 0.18). No differences in ROM were measured, with all patients achieving 170° of abduction and flexion. No SSDs were detected for internal and external rotation. Patients almost reached their pre-injury sport levels, with no significant difference between the groups (MIOP 3.9 ± 0.4 vs. AR 3,9 ± 0.3, *p* = 0.36). No operative revisions were necessary in any of the treatment groups due to clinical failure or postoperative shoulder stiffness.

## Discussion

This study showed no significant clinical and sonographic differences at mid-term follow-up when using two minimally invasive ACJ reconstruction techniques. Both techniques were reliable and achieved good to excellent clinical results in a middle-aged population with moderate functional demands. All patients were able to return to their pre-injury sport levels.

In a recent consensus report of ESA-ESSKA members, an arthroscopic-assisted anatomic ACJ reconstruction using a suspensory fixation device was advocated [16]. This arthroscopic approach is very common in sports medicine and has been proven to yield good to excellent clinical outcomes. In light of a considerable rate of concomitant intra-articular injuries, the arthroscopic technique provides the distinctive advantage of diagnosis and treatment of these pathologies. In contrast, an open approach and open reduction and internal fixation using a hook plate has long been considered the gold standard in ACJ reconstruction, but recent studies have shown the superiority of minimally invasive techniques, which is in line with the recent literature about the dynamic nature of the ACJ [17–22].

Nevertheless, the mini-open approach has been described as a valuable alternative to arthroscopic-assisted techniques. It can easily be implemented as a single or double-tunnel CC reconstruction with or without AC cerclage, and it has been shown to yield comparable clinical outcomes [5, 13]. Due to high reported numbers of combined intra-articular pathologies, we recommend a setting in which the patients can be administered to a preoperative MRI within 3 weeks after trauma to rule out any intra-articular pathologies for any approach without glenohumeral arthroscopy [20, 23].

To our knowledge, only one other study exists comparing the mini-open and arthroscopic approaches. Faggiani et al. retrospectively reviewed 16 patients (8 per group) with a mean follow-up of 13 months. In their cohort, patients treated with the mini-open technique returned to their sports significantly earlier than those treated with the arthroscopic technique, whereas the latter achieved significantly better results in the objective CS parameters [24].

So far, no definite superiority of an additional AC cerclage has been clinically proven although it seems to achieve better clinical scores, which may be because it controls posterior translation and decreases rotation [25, 26]. However, the importance of the ACJ capsule has been highlighted in a cadaveric study by Dyrna et al., which is why a suture reconstruction of the capsule seems essential according to the authors [27]. However, no clinical superiority has been shown thus far.

In the end, the number and size of clavicle drill holes increase fracture risk. This is an especially important argument in patients who participate in contact sports, where a single-tunnel technique combined with AC cerclage for ACJ reduction is more favorable [28–30]. Therefore, it is recommended to drill 2.8 mm tunnels in the clavicle and spare the lateral 2 cm of the clavicle for placing the vertical tunnel [31].

ACJ dislocation is a sports-related shoulder injury, and the clinical outcome may critically depend on the patient’s age and functional demands. In comparison to other studies, patients included in our study were older and had less athletic demands, which may influence the overall clinical results and generalizability. Furthermore, the indication for an additional AC cerclage may be stronger for a specific patient population with a high risk of failure, e.g., male patients, overhead athletes, males, and young patients [7, 16].

In terms of the surgical technique used in both groups, one can estimate the biomechanical value of the different techniques by the sonographic results provided in our study. Both techniques seemed to re-establish the CC ligaments. Interestingly, there appeared to be a trend toward worse AC reduction using the double-tunnel MIOP technique. Although the medial tunnel creates a force vector that can reduce the medial-to-lateral ACJ width, it has been demonstrated biomechanically that an AC cerclage technique is more effective at restraining posterior acromioclavicular instability compared to a double-tunnel CC technique [25]. On the other hand, there are also biomechanical studies questioning the need for an additional AC cerclage in patients with a double-tunnel technique [32, 33]. Breuer et al. did not detect persistent horizontal instability in a postoperative radiological assessment when using a double-tunnel mini-open technique without AC cerclage [5]. Hence, no conclusion can be drawn based on our data, other than the fact that no impact on the clinical outcome was apparent in our mid-term clinical results.

Sonographic follow-up examination offers a valuable tool in ACJR and is already well established [34, 35]. Its absolute values may vary due to interindividual differences and the plane used for measurements. Therefore, in our opinion, measuring the SSD is much more valuable.

Our absolute values at follow-up are comparable to data in the literature, with a certain loss of reduction being known to occur with different techniques [5, 36, 37]. In line with our data, a recent systematic review could not detect significant differences between open and arthroscopic ACJR techniques in terms of loss of reduction and complication rate [37].

This study has some important limitations due to the retrospective study design and small number of patients included. The loss of follow-up was caused by a young patient population that changed their place of residence after the operation and challenges in patients’ recruitment during the COVID-19 pandemic.

In terms of ACJR technique, the arthroscopic technique was performed with an additional AC cerclage but a single-tunnel CC reconstruction, which compromised comparability. Although sonographic assessment is validated for the measurement of the ACJ, other radiological phenomena like tunnel widening and/or button migration cannot be detected without radiological assessment [38]. Furthermore, no ACJ specific clinical outcome score has been used to assess the clinical outcome; hence, potential differences may not have been detected.

## Conclusion

After mid-term follow-up, no significant clinical or sonographic differences were apparent when comparing a mini-open ACJR and an arthroscopic-assisted ACJR with additional acromioclavicular cerclage. The outcome was evaluated in an older cohort with little to no highly demanding sports activities, which should be considered when comparing these results to other studies examining high-risk patients.
